# Recurrent amplification of *RTEL1* and *ABCA13* and its synergistic effect associated with clinicopathological data of gastric adenocarcinoma

**DOI:** 10.1186/s13039-016-0260-x

**Published:** 2016-06-29

**Authors:** T. M. Araújo, A. D. Seabra, E. M. Lima, P. P. Assumpção, R. C. Montenegro, S. Demachki, R. M. Burbano, A. S. Khayat

**Affiliations:** Human Cytogenetics Laboratory, Federal University of Pará, Belém, 66075-110 Pará Brazil; Oncology Research Center, Federal University of Pará, Belém, 66073-000 Pará Brazil; Molecular Biology Department, Federal University of Paraíba, João Pessoa, 58051-900 Paraíba Brazil

**Keywords:** Gastric cancer, aCGH, *RTEL1*, *ABCA13*, Biomarkers

## Abstract

**Background:**

Despite progression in treatment of gastric cancer, prognosis of patients remains poor, in part due to the low rate of diagnosis during its early stages. This paradigm implies the necessity to identify molecular biomarkers for early gastric cancer diagnosis, as well as for disease monitoring, thus contributing to the development of new therapeutic approaches. In a previous study, performed by array-Comparative Genomic Hybridization, we described for the first time in literature recurrent amplification of *RTEL1* and *ABCA13* genes in gastric cancer. Thus, the aim of the present study was to validate recurrent amplification of *RTEL1* and *ABCA13* genes and associate CNV status with clinicopathological data.

**Findings:**

Results showed *RTEL1* and *ABCA13* amplification in 38 % of samples. Statistical analysis demonstrated that *RTEL* amplification is more common in older patients and more associated with intestinal type and *ABCA13* amplification increases the risk of lymph node metastasis and is more common in men. Co-amplification of these genes showed a significant association with advanced staging.

**Conclusions:**

aCGH is a very useful tool for investigating novel genes associated with carcinogenesis and *RTEL1* amplification may be important for the development of gastric cancer in older patients, besides being a probable event contributing for chromosomal instability in intestinal gastric carcinogenesis. *ABCA13* amplification may have age-specific function and could be considered a useful marker for predicting lymph node metastasis in resected gastric cancer patients in early stage. Lastly, *RTEL1* and *ABCA13* synergistic effect may be considered as a putative marker for advanced staging in gastric cancer patients.

## Background

Gastric cancer is the fifth most frequent type of cancer and the third cause of cancer mortality worldwide [1].

The estimate for Brazil (2016 and 2017) indicates the occurrence of about 600,000 new cases of cancer. Except for non-melanoma skin cancer (approximately 180,000 new cases), there will be about 420,000 new cases of cancer. Among all different types of cancer that affect humans, gastric cancer, excluding non-melanoma skin cancer, ranks fourth as the most frequent tumor type in men and fifth in women [2]. In the Northern Brazil, excluding non-melanoma skin cancer, gastric cancer is the second most frequent cancer among men and the fourth among women [2].

The incidence rate of gastric cancer have decreased overall in recent years, however, it remains the leading cause of cancer-related mortality in developing countries [3]. Despite progression in treatment of advanced gastric cancer, the prognosis of gastric cancer patients remains poor, in part due to the low rate of diagnosis during its early stages [4]. This paradigm implies the necessity to search and identify molecular biomarkers for early gastric cancer diagnosis, as well as for disease monitoring, thus contributing to the development of new therapeutic approaches [5].

With regard to gastric cancer treatment, with the exception of trastuzumab (therapy based on the overexpression of HER2 protein and/or the amplification of its gene *ERBB2*), chemotherapy of localized and advanced gastric cancer still does not consider genotypic tumor characteristics. This implies that part of the patients, if not the majority, receives medical treatment with suboptimal or even lacking efficacy [6, 7].

Recently, numerous studies have investigated the molecular basis of gastric cancer involving the alteration of pathogenesis, including the mechanisms of invasion and metastasis. With the development of modern technologies, various novel biomarkers had been identified that appear to possess diagnostic and prognostic value [4]. Among the types of biomarkers, copy number variation (CNV) in key genes has been found in gastric cancer [8–10].

In a previous study, preformed by array-Comparative Genomic Hybridization (aCGH), we observed many genomic alterations in 22 patients and described for the first time in literature the recurrent amplification of *RTEL1* (*Regulator of Telomere Length 1*) gene, located on 20q13.33 (OMIM: 608833), and *ABCA13* (*ATP-Binding Cassette, Sub-Family A, Member 13*) gene, located on 7p12.3 (OMIM: 607807), in gastric adenocarcinoma, with a frequency of 50 and 23 %, respectively [11].

RTEL1 is an essential helicase that has been demonstrated to be required for the maintenance of telomere length and genomic stability. Thus, RTEL1 dysfunction is dramatically mutagenic and plays an important role in tumor initiation and progression [12, 13].

*ABCA13* gene is a member of ABC (ATP-binding cassette) family of transporters that plays a crucial role in the development of resistance by the efflux of anticancer agents outside of cancer cells [14]. Recently, several studies have associated overexpression of *ABCA13* with poor prognosis of cancer [15, 16].

Therefore, the aim of the present study was to validate the recurrent amplification of *RTEL1* and *ABCA13* genes observed previously by aCGH and associate CNV status with clinicopathological data of patients.

It is noteworthy to mention that we have chosen *RTEL1* gene due to high frequency of its amplification in gastric adenocarcinoma samples observed by aCGH. On the other hand, *ABCA13* was chosen due to its significant association with serosal extravasation. For both genes, we have also taken into account the agreement of results with data present in literature.

CNV analysis demonstrated a significant association between gene amplification and co-amplification with clinicopathological data of patients with gastric adenocarcinoma.

## Results

**Fig. 1:**
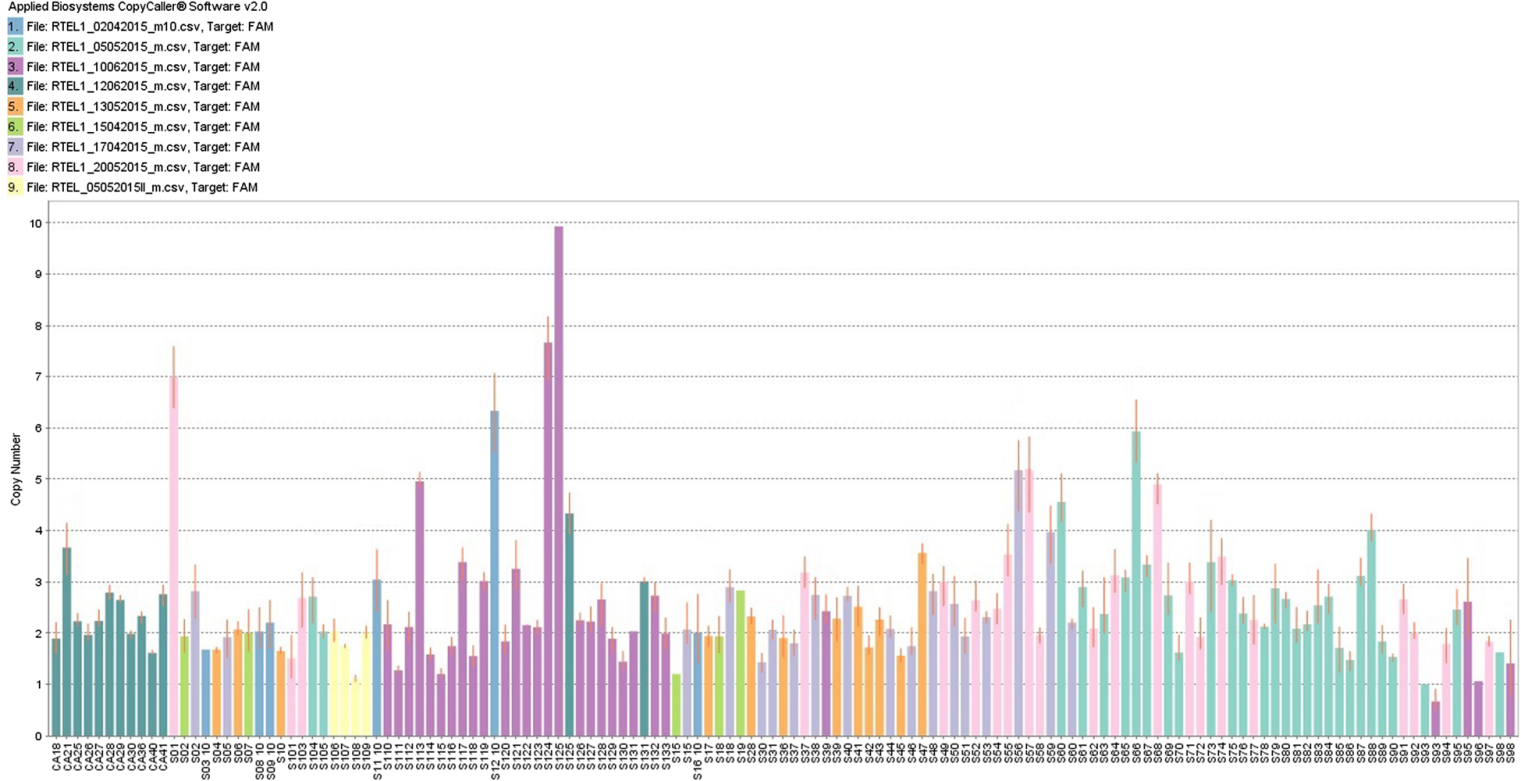
Copy number bar plot of *RTEL1* gene showing high frequency of amplification in gastric adenocarcinoma samples

*RTEL1* gene amplification was observed in 38 % of samples (Fig. 1). Statistical analysis showed that this amplification is 2.6 times more common in patients older than 50 years (*p* = 0.045; 95 % CI = 1.011–5.128) and 2.3 times more associated with intestinal type (*p* = 0.034; 95 % CI = 1.057–6.3) (Table 1).

**Table 1:** Clinicopathological data of patients with and without *RTEL1* and *ABCA13* amplification

	*RTEL1* CNV		*p-*value	*ABCA13* CNV		*p-*value	*RTEL1* and *ABCA13* amplification		*p-*value
	≥3 copies	Others		≥3 copies	Others		Present	Absent	
Gender									
Male	32	53	0.987	38	42	0.003*	17	61	0.031*
Female	15	25		7	30		2	34	
Age									
≤50 years	8	27	0.034*	10	21	0.408	2	29	0.074
>50 years	39	51		35	51		17	66	
Histopathology									
Intestinal	36	46	0.045*	34	43	0.079	15	61	0.214
Diffuse	11	32		11	29		4	34	
Localization									
Cardia	12	12	0.197	9	15	0.901	4	19	0.969
Non-cardia	35	63		35	55		15	73	
Stage									
I–II	17	37	0.218	16	34	0.215	4	43	0.05*
III–IV	30	41		29	38		15	52	
pN									
N0	9	19	0.395	5	19	0.033*	2	23	0.157
N1 or more	37	53		39	48		17	67	
pT									
T1–T3	34	62	0.359	36	53	0.431	13	74	0.375
T4	13	16		9	19		6	21	
pM									
M0	10	15	0.746	10	13	0.741	4	17	0.360
M1	12	15		13	14		8	18	

*M* male, *F* female, *pN* lymph node metastasis status, *N0* without lymph node metastasis, *N1 or more* metastasis in one ore more lymph nodes, *pT* extent of the primary tumor, *T1–T3* without serosal extravasation, *T4* with serosal extravasation, *pM*, distant metastasis status, *M1* with distant metastasis, *M0* without distant metastasis. *Significant difference between groups with and without amplification, *p* ≤ 0.05, Chi-square test

**Fig. 2:**
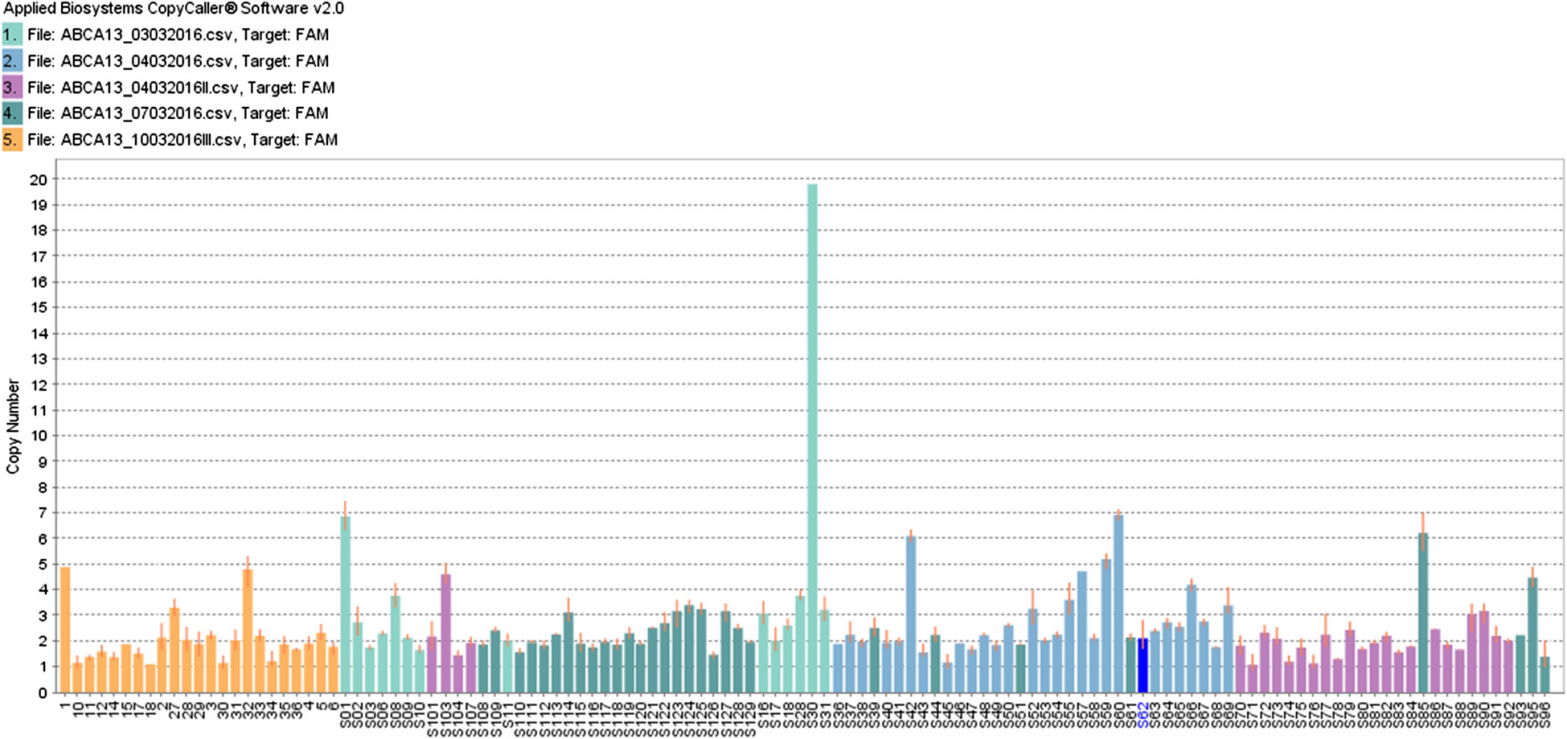
Copy number bar plot of *ABCA13* gene showing high frequency of amplification in gastric adenocarcinoma samples

*ABCA13* gene amplification was also observed in 38 % of samples (Fig. 2). Statistical analysis showed that *ABCA13* amplification increases 3 times the risk of lymph node metastasis (*p* = 0.033; 95 % CI = 1.057–9.018). Additionally, this amplification is 4 times more common in men (*p* = 0.003; 95 % CI = 1.526–9.851) and demonstrated an inconclusive association with intestinal type (*p* = 0.079) (Table 1).

Synergistic effect of these two amplifications was also evaluated. The results showed that *RTEL1* and *ABCA13* amplification in the same patient was 3 times more associated with advanced stage (III and IV) (*p* = 0.05; 95 % CI = 0.958–10.037) (Table 1).

Subcategorization of samples into intestinal and diffuse types did not result in any significant clinicopathological association.

It is important to note that 51 % (23/45) and 21 % (10/47) of *ABCA13* and *RTEL1* amplifications, respectively, were high-level amplifications (≥5 copies), but subdivision of samples into high-level amplifications and other amplifications (3 and 4 copies) or high-level amplifications and all other (1, 2, 3 and 4 copies) did not result in any significant clinicopathological association.

Regarding oral squamous cell carcinoma, we observed 30 and 25 % of *RTEL1* and *ABCA13* amplification, respectively, but the presence of amplification was not statistically associated with clinicopathological data of patients.

## Discussion

High amplification frequencies observed in the present study corroborate with the previous study performed by aCGH, indicating that this technique is useful to investigate CNV and find novel genes associated with diseases, even with a low number of cases.

The statistical association of *ABCA13* with serosal extravasation [11] was not confirmed by real time PCR and new significant associations were observed, including for *RTEL1* gene. This discrepancy is probably due to differences in sample size of each investigation.

The region 20q, where *RTEL1* gene is located, is amplified in several types of cancer [17–19], but we were the first group to describe *RTEL1* amplification in gastric cancer [11].

Rtel1 is an essential helicase for telomere maintenance and the regulation of homologous recombination (HR) [12]. HR is one of the major pathways to maintain genomic stability and is involved in the repair of complex DNA damage, DSBs, interstrand crosslinks and DNA gaps. Thus, upregulated Rtel1 function might prevent HR when it is needed as a legitimate means for repair, leading to malfunction of repair system [20].

Another hypothesis is that the excessive activity of Rtel1 would increase Rtel1 helicase activity, leading to disengaging of T-loop structure and, consequently, to telomere deprotection, which may result in end-to-end fusions and exonucleolytic attack [13, 21].

Several observations suggested a possible role for *RTEL1* during DNA replication. Mouse cells deficient for *RTEL1* exhibit reduced proliferative capacity, and worms and mammalian cells lacking *RTEL1* are particularly sensitive to DNA damaging agents that hinder DNA replication, such as inter-strand crosslinking agents [13, 20, 22].

Wu et al. [12] showed that increased expression of Rtel1 in mouse hepatocytes induced the development of liver tumors. This finding is consistent with human genetic data that showed that amplification of *RTEL1* genomic locus is not only a common genetic alteration in human hepatocellular carcinoma, but also closely associated with its malignancy and progression [23–27].

In this study, we found for the first time recurrent *RTEL1* amplification statistically associated with advanced age and intestinal type of gastric adenocarcinoma. Thus, we suggest that amplification of *RTEL1* may have age-specific function and an important role in adenocarcinoma of intestinal type, which corroborates with the hypothesis that these two histological types have different genetic pathways [28, 29].

Consistent with these results, El-Rifai et al. [30] and Kokkola et al. [31] found a significant association between 20q amplification and intestinal type of gastric cancer. Interestingly, a molecular classification of gastric cancer, proposed by Cancer Genome Atlas Research Network [32], categorized intestinal gastric cancer as correlated with chromosomal instability. Thus, *RTEL1* may be a key gene of 20q region, since its upregulation triggers chromosomal instability [13, 21].

Noteworthy, we observed that the frequency of *RTEL1* amplification is almost equal in all stages of cancer (I-41 %, II-30 %, III-44 % and IV-44 %), corroborating with the hypothesis proposed by Tabach et al. [33] that amplification of the 20q chromosomal arm occurs early in tumorigenic transformation and may initiate cancer.

The human ABC transporters are encoded by a large transporter gene superfamily, which is composed of 49 members grouped into seven subfamilies (A–G) according to the sequence homology. ABC proteins facilitate translocation of heterogeneous substrates including metabolic products, lipids and sterols, peptides and proteins, saccharides, amino acids and drugs across the cell membrane. To transport these substrates across extracellular and intracellular membranes against a concentration gradient, ABCs use energy acquired by the hydrolysis of ATP [34].

*ABCA13* is a member of ABC gene subfamily A (ABCA) that plays a crucial role in the development of resistance by the efflux of anticancer agents outside of cancer cells [14] and overexpression of one or more membrane-bound ATP-binding cassette (ABC) transportes has been associated with such mechanism of drug resistance [35].

There are few studies in literature regarding the role of *ABCA13* in cancer, but they demonstrate a positive association between *ABCA13* upregulation and unfavorable outcomes.

Upregulation of *ABCA12*, *ABCA13*, *ABCB6*, *ABCC1*, *ABCC2* and *ABCE1* genes was found by Hlavata et al. [14] in colorectal cancer samples when compared to normal tissues and Nymoen et al. [15] observed that *ABCA13* mRNA overexpression was significantly related to shorter overall survival in metastatic ovarian serous carcinoma.

Importantly, Hlaváč et al. [16] stated that *ABCA13*, *ABCB3* and *ABCC1* levels were significantly higher in patients with grade 3 than in patients with grade 1 or 2 of breast carcinoma, suggesting that overexpression of these genes may be associated with poor prognosis.

In the present study, we found for the first time recurrent amplification of *ABCA13* statistically associated with lymph node metastasis in gastric carcinogenesis. Therefore, we suggest that amplification of *ABCA13* gene has an important role in development of lymph node metastasis, which is associated with poor outcomes [36].

Moreover, we observed a significant association between *ABCA13* amplification and male gender. In this regard, it is important to note that ABC transporters can move substrates in (influx) or out (efflux) of cells, such as inorganic anions, metal ions and a large number of hydrophobic compounds and metabolites across the plasma membrane [37], which may include substances that could be harmful to health. Since men are more exposed to carcinogens than woman [38–43], the influx of carcinogenic substances by *ABCA13* channel could explain the high frequency of amplification of this gene in male gender related to gastric cancer.

## Conclusion

aCGH is a very useful tool for investigating novel genes associated with carcinogenesis. Through this technique, we were able to identify recurrent amplification of *RTEL1* and *ABCA13* and this observation was validated by real-time PCR for copy number analysis on a larger number of samples and in other type of cancer, demonstrating that these genes may have important roles in the carcinogenesis process. Thus, *RTEL1* amplification may be important for the development of gastric cancer in older patients, besides being a probable event contributing for chromosomal instability in intestinal gastric carcinogenesis. Moreover, *ABCA13* amplification may have age-specific function and could be considered a useful marker for predicting lymph node metastasis in resected gastric cancer patients in early stage. Lastly, *RTEL1* and *ABCA13* synergistic effect may be considered as a putative marker for advanced staging in gastric cancer patients.

## Methods

### Samples

We analyzed gastric adenocarcinoma samples obtained from primary gastric tumors of patients from João de Barros Barreto University Hospital (HUJBB), located in Pará State, Brazil.

All samples were obtained before administration of chemical treatments or radiotherapy. This study was approved by HUJBB ethics committee (CAAE: 42999115.7.0000.5634) and all individuals signed a Consent Form allowing the use of biological samples and clinical data.

For *RTEL1* copy number investigation we used 125 fresh frozen samples (Table 1), 68 % from male and 32 % from female patients, with a mean age of 59 years (±13). Regarding tumor site, 80 % were obtained from tumors located outside from cardia region and 20 % of tumors located in the cardia region. Of the total of samples, 57 % were collected from patients with advanced stage (III and IV) and 43 % from patients with early stage (I and II), 66 % of tumors belonging to the intestinal type and 34 % to the diffuse type of Laurén. Also with respect to staging, it was observed that 23 % of patients presented serosal extravasation (T4), 76 % presented lymph node metastasis and 52 % presented distant metastasis. For *ABCA13* copy number investigation we used 117 fresh frozen samples (Table 1), because eight reactions were unsuccessful.

Additionally, we evaluated CNV status of these genes in 47 oral squamous cell carcinoma samples, in an attempt to investigate if the frequency of *RTEL1* and *ABCA13* amplification is also high in other type of neoplasia. Sample composition was 64 % male and 36 % female patients, with a mean age of 61 years (±13). Regarding tumor site, 80 % were obtained from tumors located in tongue or floor of mouth. Of the total of samples, 56 % were collected from patients with advanced stage (III and IV) and 44 % from patients with early stage (I and II).

### Histopathology

Tumor samples were included only if at least 80 % of the sample consisted of cancer cells. Histopathological data, such as histological subtype, degree of differentiation, depth of invasion, lymph node involvement and distant metastasis were taken from pathology reports of the Department of Pathology of HUJBB. The histopathological analysis of tumor fragments was performed according to Laurén’s classification [44].

### Quantitative analysis of copy number variants based on real-time PCR

Genomic DNA extraction was carried out using Gentra Puregene Kit (Qiagen, Germantown, MD, USA), according to the manufacturer’s instructions. After extraction, we evaluated quantity and quality of each sample using Nanodrop 1000 Spectophotometer (NanoDrop Technologies, Houston, TX, USA). The volume for each sample was adjusted accordingly to achieve 10 ng/μl using nuclease free water.

We performed TaqMan Copy Number Assay (Applied Biosystems, Foster City, CA). Briefly, 1 μl of 10 ng DNA was added to 5 μL of TaqMan Genotyping Master Mix (Applied Biosystems, Foster City, CA), with 0.5 μl of *RTEL1* or *ABCA13* probe and 3 μL of water. We measured copy number gain using the following profile: denaturation at 95 °C for 10 min, followed by 40 cycles of 95 °C for 15 s and 60 °C for 1 min. We determined relative quantification using the 7500 Rreal-time PCR system (Applied Biosystems, Foster City, CA) in quadruplicate. RNaseP (Applied Biosystems, Foster City, CA) was used as a control. After amplification, we imported the experiment results containing threshold-cycle values for the copy number and reference assay into the CopyCaller Software (Applied Biosystems, Foster City, CA) for post-PCR data analysis as previously described by Graziano et al. [45].

### Statistics

Statistical analysis for comparisons of categorical variables between groups were done by means of Chi-square test and were performed using PASW Statistics program. Odds Ratio (OR) and Confidence Interval (CI = 95 %) were also calculated. A two-tailed probability value *p* ≤ 0.05 was considered to be statistically significant.

## Abbreviations

ABC, ATP-binding cassette; *ABCA12*, *ATP-Binding Cassette, Sub-Family A, Member 12; ABCA13*, *ATP-Binding Cassette, Sub-Family A, Member 13; ABCB3*, *ATP-Binding Cassette, Sub-Family B, Member 3; ABCB6*, *ATP-Binding Cassette, Sub-Family B, Member 6; ABCC1*, *ATP-Binding Cassette, Sub-Family C, Member 1; ABCC2*, *ATP-Binding Cassette, Sub-Family C, Member 2; ABCE1*, *ATP-Binding Cassette, Sub-Family E, Member 1;* aCGH, array Comparative Genomic Hybridization; CNV, copy number variation; DSBs, Double Strand Breaks; *ERBB2*, *Erb-B2 Receptor Tyrosine Kinase 2;* HER2, human epidermal growth factor receptor 2; HR, homologous recombination; HUJBB, João de Barros Barreto University Hospital; PCR, polymerase chain reaction; *RTEL1*, *Regulator of Telomere Length 1*

## References

[1] Ferlay J, Soerjomataram I, Ervik M, Dikshit R, Eser S, Mathers C, Rebelo M, Parkin DM, Forman D, Bray F (2013). GLOBOCAN 2012 v1.0, Cancer Incidence and Mortality Worldwide: IARC CancerBase No. 11 [Internet].

[2] INCA ‐ Instituto Nacional do Câncer. Estimativa 2016/2017- Incidência de câncer no Brasil. http://www.inca.gov.br. Acessed 4 mar 2016.

[3] Jemal A, Bray F, Center MM (2011). Global cancer statistics. CA Cancer J Clin.

[4] Jin Z, Jiang W, Wang L (2015). Biomarkers for gastric cancer: progression in early diagnosis and prognosis (Review). Oncol Lett.

[5] Wu HH, Lin WC, Tsai KW (2014). Advances in molecular biomarkers for gastric cancer: miRNAs as emerging novel cancer markers. Expert Rev Mol Med.

[6] Warneke VS, Behrens HM, Haag J, Balschun K, Böger C, Becker T, Ebert MP, Lordick F, Röcken C (2013). Prognostic and putative predictive biomarkers of gastric cancer for personalized medicine. Diagn Mol Pathol.

[7] Bang YJ, Van Cutsem E, Feyereislova A, Chung HC, Shen L, Sawaki A, Lordick F, Ohtsu A, Omuro Y, Satoh T, Aprile G, Kulikov E, Hill J, Lehle M, Rüschoff J, Kang YK, ToGA Trial Investigators (2010). Trastuzumab in combination with chemotherapy versus chemotherapy alone for treatment of HER2-positive advanced gastric or gastro-oesophageal junction cancer (ToGA): a phase 3, open-label, randomised controlled trial. Lancet.

[8] Buffart TE, Carvalho B, Mons T, Reis RM, Moutinho C, Silva P, van Grieken NC, Vieth M, Stolte M, van de Velde CJ, Schrock E, Matthaei A, Ylstra B, Carneiro F, Meijer GA (2007). DNA copy number profiles of gastric cancer precursor lesions. BMC Genomics.

[9] Kang JU, Kang JJ, Kwon KC, Park JW, Jeong TE, Noh SM, Koo SH (2006). Genetic alterations in primary gastric carcinomas correlated with clinicopathological variables by array comparative genomic hybridization. J Korean Med Sci.

[10] Weiss MM, Kuipers EJ, Postma C, Snijders AM, Pinkel D, Meuwissen SG, Albertson D, Meijer GA (2004). Genomic alterations in primary gastric adenocarcinomas correlate with clinicopathological characteristics and survival. Cell Oncol.

[11] Seabra AD, Araújo TM, Mello Junior FA, Di Felipe Ávila Alcântara D, De Barros AP, De Assumpção PP, Montenegro RC, Guimarães AC, Demachki S, Burbano RM, Khayat AS (2014). High-density array comparative genomic hybridization detects novel copy number alterations in gastric adenocarcinoma. Anticancer Res.

[12] Wu X, Sandhu S, Nabi Z, Ding H (2012). Generation of a mouse model for studying the role of upregulated RTEL1 activity in tumorigenesis. Transgenic Res.

[13] Barber LJ, Youds JL, Ward JD, McIlwraith MJ, O’Neil NJ, Petalcorin MI, Martin JS, Collis SJ, Cantor SB, Auclair M, Tissenbaum H, West SC, Rose AM, Boulton SJ (2008). RTEL1 maintains genomic stability by suppressing homologous recombination. Cell.

[14] Hlavata I, Mohelnikova-Duchonova B, Vaclavikova R, Liska V, Pitule P, Novak P, Bruha J, Vycital O, Holubec L, Treska V, Vodicka P, Soucek P (2012). The role of ABC transporters in progression and clinical outcome of colorectal cancer. Mutagenesis.

[15] Nymoen DA, Holth A, Hetland Falkenthal TE, Tropé CG, Davidson B. CIAPIN1 and ABCA13 are markers of poor survival in metastatic ovarian serous carcinoma. Mol Cancer. doi: 10.1186/s12943-015-0317-1. 10.1186/s12943-015-0317-1 PMC4336750 25889687

[16] Hlaváč V, Brynychová V, Václavíková R, Ehrlichová M, Vrána D, Pecha V, Koževnikovová R, Trnková M, Gatěk J, Kopperová D, Gut I, Souček P (2013). The expression profile of ATP-binding cassette transporter genes in breast carcinoma. Pharmacogenomics.

[17] Gorringe KL, Hunter SM, Pang JM, Opeskin K, Hill P, Rowley SM, Choong DY, Thompson ER, Dobrovic A, Fox SB, Mann GB, Campbell IG (2015). Copy number analysis of ductal carcinoma in situ with and without recurrence. Mod Pathol.

[18] Scotto L, Narayan G, Nandula SV, Arias-Pulido H, Subramaniyam S, Schneider A, Kaufmann AM, Wright JD, Pothuri B, Mansukhani M, Murty VV (2008). Identification of copy number gain and overexpressed genes on chromosome arm 20q by an integrative genomic approach in cervical cancer: potential role in progression. Genes Chromosomes Cancer.

[19] Burbano RR, Assumpção PP, Leal MF, Calcagno DQ, Guimarães AC, Khayat AS, Takeno SS, Chen ES, De Arruda Cardoso Smith M (2006). C-MYC locus amplification as metastasis predictor in intestinal-type gastric adenocarcinomas: CGH study in Brazil. Anticancer Res.

[20] Uringa EJ, Youds JL, Lisaingo K, Lansdorp PM, Boulton SJ (2011). RTEL1: an essential helicase for telomere maintenance and the regulation of homologous recombination. Nucleic Acids Res.

[21] Bailey SM, Murnane JP (2006). Telomeres, chromosome instability and cancer. Nucleic Acids Res.

[22] Ding H, Schertzer M, Wu X, Gertsenstein M, Selig S, Kammori M, Pourvali R, Poon S, Vulto I, Chavez E, Tam PP, Nagy A, Lansdorp PM, Niketeghad F, Decker HJ, Caselmann WH, Lund P, Geissler F, Dienes HP, Schirmacher P (2004). Regulation of murine telomere length by Rtel: an essential gene encoding a helicase-like protein. Cell.

[23] Taniguchi K, Yamada T, Sasaki Y, Kato K (2010). Genetic and epigenetic characteristics of human multiple hepatocellular carcinoma. BMC Cancer.

[24] Katoh H, Shibata T, Kokubu A, Ojima H, Fukayama M, Kanai Y, Hirohashi S (2006). Epigenetic instability and chromosomal instability in hepatocellular carcinoma. Am J Pathol.

[25] Niketeghad F, Decker HJ, Caselmann WH, Lund P, Geissler F, Dienes HP, Schirmacher P (2001). Frequent genomic imbalances suggest commonly altered tumour genes in human hepatocarcinogenesis. Br J Cancer.

[26] Guan XY, Fang Y, Sham JS, Kwong DL, Zhang Y, Liang Q, Li H, Zhou H, Trent JM. Recurrent chromosome alterations in hepatocellular carcinoma detected by comparative genomic hybridization.Genes Chromosomes Cancer. 2000. doi: 10.1002/1098-2264(2000)9999:9999%3C::AID-GCC1022%3E3.0.CO;2-V. 11107185

[27] Wong N, Lai P, Lee SW, Fan S, Pang E, Liew CT, Sheng Z, Lau JW, Johnson PJ (1999). Assessment of genetic changes in hepatocellular carcinoma by comparative genomic hybridization analysis: relationship to disease stage, tumor size, and cirrhosis. Am J Pathol.

[28] Tahara E (2004). Genetic pathways of two types of gastric cancer. IARC Sci Publ.

[29] Yasui W, Yokozaki H, Fujimoto J, Naka K, Kuniyasu H, Tahara E (2000). Genetic and epigenetic alterations in multistep carcinogenesis of the stomach. J Gastroenterol.

[30] El-Rifai W, Harper JC, Cummings OW, Hyytinen ER, Frierson HF, Knuutila S, Powell SM (1998). Consistent genetic alterations in xenografts of proximal stomach and gastro-esophageal junctionadenocarcinomas. Cancer Res.

[31] Kokkola A, Monni O, Puolakkainen P, Larramendy ML, Victorzon M, Nordling S, Haapiainen R, Kivilaakso E, Knuutila S (1997). 17q12-21 amplicon, a novel recurrent genetic change in intestinal type of gastric carcinoma: a comparative genomic hybridization study. Genes Chromosomes Cancer.

[32] Cancer Genome Atlas Research Network. Comprehensive molecular characterization of gastric adenocarcinoma. Nature. 2014. doi: 10.1038/nature13480. 10.1038/nature13480 PMC4170219 25079317

[33] Tabach Y, Kogan-Sakin I, Buganim Y, Solomon H, Goldfinger N, Hovland R, Ke XS, Oyan AM, Kalland KH, Rotter V, Domany E (2011). Amplification of the 20q chromosomal arm occurs early in tumorigenic transformation and may initiate cancer. PLoS One.

[34] Higgins CF (1992). ABC transporters: from microorganisms to man. Annu Rev Cell Biol.

[35] Szakács G, Paterson JK, Ludwig JA, BoothGenthe C, Gottesman MM (2006). Targeting multidrug resistance in cancer. Nat Rev Drug Discov.

[36] Deng JY, Liang H (2014). Clinical significance of lymph node metastasis in gastric cancer. World J Gastroenterol.

[37] Vasiliou V, Vasiliou K, Nebert DW (2009). Human ATP-binding cassette (ABC) transporter Family. Hum Genomics.

[38] Freedman ND, Abnet CC, Leitzmann MF, Mouw T, Subar AF, Hollenbeck AR, Schatzkin A (2007). A prospective study of tobacco, alcohol, and the risk of esophageal and gastric cancer subtypes. Am J Epidemiol.

[39] Kirsch-Volders M, Bonassi S, Herceg Z, Hirvonen A, Möller L, Phillips DH (2010). Gender-related differences in response to mutagens and carcinogens. Mutagenesis.

[40] Kadekar S, Peddada S, Silins I, French JE, Högberg J, Stenius U (2012). Gender differences in chemical carcinogenesis in National Toxicology Program 2-year bioassays. Toxicol Pathol.

[41] Saghier AA, Kabanja JH, Afreen S, Sagar M (2013). Gastric cancer: environmental risk factors, treatment and prevention. J Carcinogene Mutagene.

[42] Cocco P, Ward MH, Buiatti E (1996). Occupational risk factors for gastric cancer: an overview. Epidemiol Rev.

[43] Borugian MJ, Lee TK, Black P, Bressler B, Goldenberg SL, Gallagher R. Cancer among males in BC and Canada. BCMJ. 2011;53(10):541–46.

[44] Laurén P (1965). The two histological main types of gastric carcinoma: diffuse and so‐called intestinal‐type carcinoma. An attempt at a histo‐clinical classification. Acta Pathol Microbiol Scand.

[45] Graziano F, Galluccio N, Lorenzini P, Ruzzo A, Canestrari E, D’Emidio S, Catalano V, Sisti V, Ligorio C, Andreoni F, Rulli E, Di Oto E, Fiorentini G, Zingaretti C, De Nictolis M, Cappuzzo F, Magnani M (2011). Genetic activation of the MET pathway and prognosis of patients with high-risk, radically resected gastric cancer. J Clin Oncol.

